# 2-(4-Chloro­phen­yl)-4-oxo-4-phenyl­butane­nitrile

**DOI:** 10.1107/S1600536814002335

**Published:** 2014-02-08

**Authors:** Ben Ma, Hongyan Zhou, Jingya Yang

**Affiliations:** a College of Chemistry and Chemical Engineering, Northwest Normal University, Lanzhou 730070, People’s Republic of China; b College of Science, Gansu Agricultural University, Lanzhou 730070, People’s Republic of China

## Abstract

The title mol­ecule, C_16_H_12_ClNO, has a V-shaped conformation and the dihedral angle between the planes of the phenyl and benzene rings of 64.6 (1)°. No directional intermolecular interactions could be identified in the crystal.

## Related literature   

For hydro­cyanation reactions used for the synthesis of related nitrile derivatives, see: Li *et al.* (2012 ▶); Lin *et al.* (2012 ▶); Yang, Shen & Chen (2010 ▶); Yang, Wu & Chen (2010 ▶). For related structures, see: Yang *et al.* (2011 ▶); Abdel-Aziz *et al.* (2012*a* ▶, 2012*b* ▶). For nitrile-containing pharmaceuticals, see: Fleming *et al.* (2010 ▶).
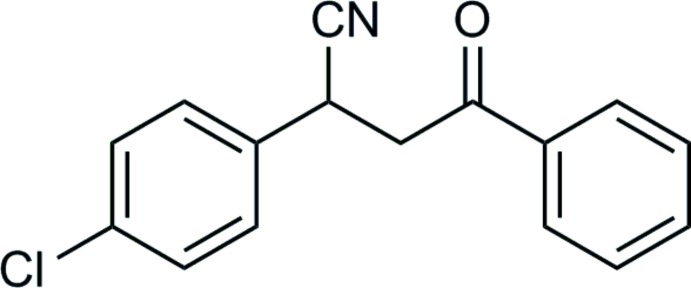


## Experimental   

### 

#### Crystal data  

C_16_H_12_ClNO
*M* *_r_* = 269.72
Orthorhombic, 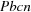

*a* = 31.247 (13) Å
*b* = 9.1889 (10) Å
*c* = 9.3719 (12) Å
*V* = 2690.9 (12) Å^3^
*Z* = 8
Mo *K*α radiation
μ = 0.27 mm^−1^
*T* = 293 K
0.44 × 0.39 × 0.37 mm

#### Data collection  

Agilent SuperNova (Dual, Cu at zero, Eos) diffractometer
Absorption correction: multi-scan (*CrysAlis PRO*; Agilent, 2013 ▶) *T*_min_ = 0.584, *T*_max_ = 1.000
6683 measured reflections
2642 independent reflections
1605 reflections with *I* > 2σ(*I*)
*R*_int_ = 0.044

#### Refinement  

*R*[*F* ^2^ > 2σ(*F* ^2^)] = 0.056
*wR*(*F* ^2^) = 0.145
*S* = 1.08
2642 reflections
172 parameters
H-atom parameters constrained
Δρ_max_ = 0.14 e Å^−3^
Δρ_min_ = −0.23 e Å^−3^

### 

Data collection: *CrysAlis PRO* (Agilent, 2013 ▶); cell refinement: *CrysAlis PRO*; data reduction: *CrysAlis PRO*; program(s) used to solve structure: *SHELXS2013* (Sheldrick, 2008 ▶); program(s) used to refine structure: *SHELXL2013* (Sheldrick, 2008 ▶); molecular graphics: *SHELXTL* (Sheldrick, 2008 ▶); software used to prepare material for publication: *OLEX2* (Dolomanov *et al.*, 2009 ▶).

## Supplementary Material

Crystal structure: contains datablock(s) I, exp_1126_4. DOI: 10.1107/S1600536814002335/bh2493sup1.cif

Structure factors: contains datablock(s) I. DOI: 10.1107/S1600536814002335/bh2493Isup2.hkl

Supporting information file. DOI: 10.1107/S1600536814002335/bh2493Isup3.cdx

Supporting information file. DOI: 10.1107/S1600536814002335/bh2493Isup4.cml

CCDC reference:

Additional supporting information:  crystallographic information; 3D view; checkCIF report

## Figures and Tables

**Fig. 1.**
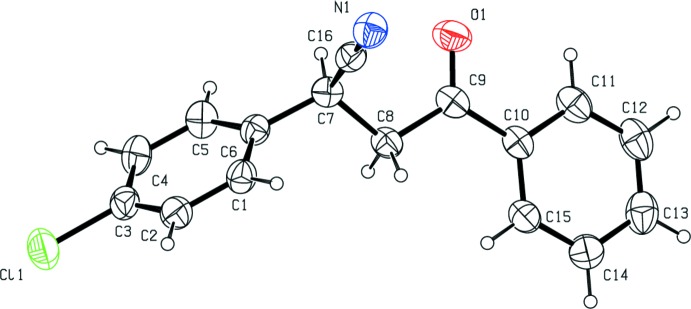
Thermal ellipsoid plot of the title compound at the 30% probability level; hydrogen atoms are drawn as spheres of arbitrary radius.

**Fig. 2.**
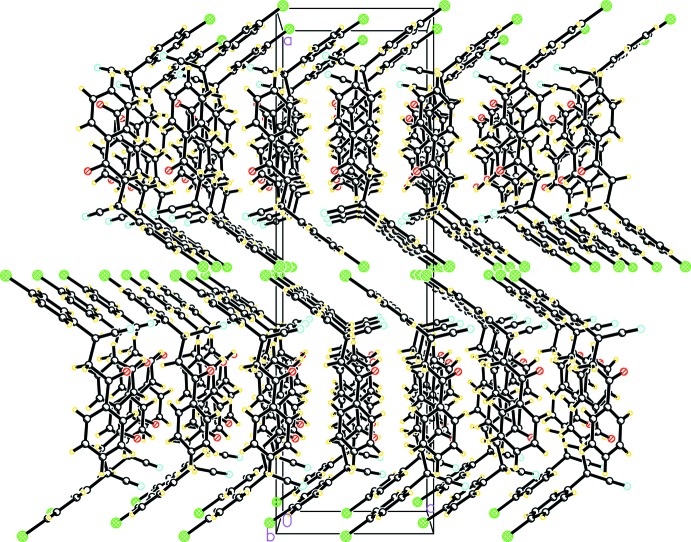
Packing diagram of the title compound.

## supplementary crystallographic information

### 1. Comment

Nitriles usually exhibit important biological and pharmacological activity. For instance, many nitrile-containing pharmaceuticals are widely used in clinical treatments (Fleming *et al.*, 2010). In addition, nitrile derivatives are essential synthetic intermediates in organic synthesis because of their easy achievements and versatile transformations (*e.g*. Li *et al.*, 2012; Lin *et al.*, 2012; Yang, Shen & Chen, 2010; Yang, Wu & Chen, 2010). The title compound exhibits a V-shaped configuration (Fig. 1), previously observed in related structures (Yang *et al.*, 2011; Abdel-Aziz *et al.*, 2012*a*, 2012*b*). One molecule interpenetrates with other symmetry-related molecules in the crystal, to generate a two-dimensional roof-like crystal structure (Fig. 2). Finally, the roof-like structures pack to be the stable crystal structure.

### 2. Experimental

The synthesis follows that previously published (Yang, Shen & Chen, 2010). After Cs_2_CO_3_ (0.5 mg, 0.0015 mmol), (*E*)-3-(4-chlorophenyl)-1-phenylprop-2-en-1-one (72.8 mg, 0.3 mmol), and dioxane (0.5 ml) were charged into a dry Schlenk tube equipped with cold finger, Me_3_SiCN (57 ml, 0.45 mmol) and H_2_O (22 ml, 1.2 mmol) were added. The reaction mixture was refluxed until the reaction was complete (as monitored by TLC). Then, H_2_O (2 ml) was added at room temperature and the resulting mixture was extracted with EtOAc (5 ml). The extract was washed with H_2_O (2 ml), brine (3 ml), dried (Na_2_SO_4_), and concentrated. The crude product was purified by flash column chromatography on silica gel (PE–EtOAc, 15:1) to afford the pure title compound as a white solid (71.2 mg, 88% yield). Colorless single crystals of the title compound suitable for X-ray structure determination were obtained by vapor diffusion of petroleum ether into an ethyl acetate solution, at room temperature.

### 3. Refinement

Carbon-bound H-atoms were placed in calculated positions (aromatic CH: 0.93 Å; methylene CH_2_: 0.97 Å; methine CH: 0.98 Å) and were included in the riding model approximation, with *U*_iso_(H) set to 1.2*U*_eq_(carrier C).

### Crystal data

**Table:**

C_16_H_12_ClNO	*D*_x_ = 1.332 Mg m^−^^3^
*M**_r_* = 269.72	Melting point: 383 K
Orthorhombic, *P**b**c**n*	Mo *K*α radiation, λ = 0.71073 Å
Hall symbol: -P 2n 2ab	Cell parameters from 1186 reflections
*a* = 31.247 (13) Å	θ = 3.7–22.6°
*b* = 9.1889 (10) Å	µ = 0.27 mm^−^^1^
*c* = 9.3719 (12) Å	*T* = 293 K
*V* = 2690.9 (12) Å^3^	Block, colourless
*Z* = 8	0.44 × 0.39 × 0.37 mm
*F*(000) = 1120	

### Data collection

**Table:**

Agilent SuperNova (Dual, Cu at zero, Eos) diffractometer	2642 independent reflections
Radiation source: MoKa	1605 reflections with *I* > 2σ(*I*)
Mirror monochromator	*R*_int_ = 0.044
Detector resolution: 16.0733 pixels mm^-1^	θ_max_ = 26.0°, θ_min_ = 3.2°
ω scans	*h* = −20→38
Absorption correction: multi-scan (*CrysAlis PRO*; Agilent, 2013)	*k* = −11→10
*T*_min_ = 0.584, *T*_max_ = 1.000	*l* = −11→11
6683 measured reflections	

### Refinement

**Table:**

Refinement on *F*^2^	0 constraints
Least-squares matrix: full	Primary atom site location: structure-invariant direct methods
*R*[*F*^2^ > 2σ(*F*^2^)] = 0.056	Hydrogen site location: inferred from neighbouring sites
*wR*(*F*^2^) = 0.145	H-atom parameters constrained
*S* = 1.08	*w* = 1/[σ^2^(*F*_o_^2^) + (0.0418*P*)^2^ + 0.4921*P*] where *P* = (*F*_o_^2^ + 2*F*_c_^2^)/3
2642 reflections	(Δ/σ)_max_ < 0.001
172 parameters	Δρ_max_ = 0.14 e Å^−^^3^
0 restraints	Δρ_min_ = −0.23 e Å^−^^3^

### Fractional atomic coordinates and isotropic or equivalent isotropic displacement parameters (Å^2^)

**Table:**

	*x*	*y*	*z*	*U*_iso_*/*U*_eq_	
Cl1	0.49185 (3)	0.22108 (11)	0.94462 (10)	0.0911 (4)	
O1	0.31808 (7)	0.4639 (2)	1.6227 (2)	0.0802 (7)	
N1	0.39810 (8)	0.1944 (3)	1.6798 (3)	0.0696 (7)	
C1	0.41798 (9)	0.1701 (3)	1.2875 (3)	0.0602 (8)	
H1	0.4064	0.0933	1.3392	0.072*	
C2	0.44356 (9)	0.1413 (3)	1.1702 (3)	0.0655 (8)	
H2	0.4492	0.0457	1.1436	0.079*	
C3	0.46042 (8)	0.2544 (4)	1.0936 (3)	0.0632 (8)	
C4	0.45302 (9)	0.3961 (4)	1.1351 (3)	0.0705 (9)	
H4	0.4652	0.4728	1.0846	0.085*	
C5	0.42749 (9)	0.4235 (3)	1.2521 (3)	0.0655 (8)	
H5	0.4224	0.5192	1.2796	0.079*	
C6	0.40944 (8)	0.3116 (3)	1.3289 (3)	0.0528 (7)	
C7	0.37986 (8)	0.3464 (3)	1.4528 (3)	0.0541 (7)	
H7	0.3839	0.4493	1.4768	0.065*	
C8	0.33248 (8)	0.3254 (3)	1.4156 (3)	0.0561 (7)	
H8A	0.3269	0.3682	1.3228	0.067*	
H8B	0.3263	0.2221	1.4094	0.067*	
C9	0.30306 (10)	0.3939 (3)	1.5247 (3)	0.0579 (7)	
C10	0.25590 (9)	0.3757 (3)	1.5110 (3)	0.0541 (7)	
C11	0.22948 (10)	0.4530 (3)	1.6019 (3)	0.0699 (9)	
H11	0.2415	0.5151	1.6692	0.084*	
C12	0.18561 (11)	0.4396 (4)	1.5944 (4)	0.0797 (10)	
H12	0.1683	0.4923	1.6563	0.096*	
C13	0.16764 (11)	0.3486 (4)	1.4955 (4)	0.0809 (10)	
H13	0.1380	0.3393	1.4904	0.097*	
C14	0.19339 (11)	0.2709 (4)	1.4037 (4)	0.0770 (9)	
H14	0.1812	0.2091	1.3364	0.092*	
C15	0.23731 (10)	0.2848 (3)	1.4116 (3)	0.0643 (8)	
H15	0.2546	0.2323	1.3493	0.077*	
C16	0.39056 (9)	0.2607 (3)	1.5808 (3)	0.0559 (7)	

### Atomic displacement parameters (Å^2^)

**Table:**

	*U*^11^	*U*^22^	*U*^33^	*U*^12^	*U*^13^	*U*^23^
Cl1	0.0720 (5)	0.1253 (8)	0.0759 (6)	0.0063 (5)	0.0188 (5)	−0.0007 (5)
O1	0.0793 (14)	0.0887 (16)	0.0725 (14)	0.0126 (12)	−0.0085 (12)	−0.0260 (12)
N1	0.0627 (15)	0.0749 (18)	0.0712 (18)	0.0033 (13)	−0.0059 (14)	0.0092 (14)
C1	0.0579 (16)	0.0528 (17)	0.070 (2)	−0.0036 (14)	0.0068 (16)	0.0033 (14)
C2	0.0604 (17)	0.0644 (18)	0.072 (2)	0.0008 (16)	0.0029 (17)	−0.0078 (16)
C3	0.0445 (15)	0.085 (2)	0.0606 (19)	0.0018 (15)	−0.0017 (15)	0.0050 (16)
C4	0.0598 (17)	0.072 (2)	0.080 (2)	−0.0025 (16)	0.0099 (17)	0.0201 (17)
C5	0.0653 (17)	0.0531 (17)	0.078 (2)	0.0058 (15)	0.0081 (17)	0.0083 (15)
C6	0.0490 (14)	0.0547 (16)	0.0548 (17)	−0.0010 (13)	−0.0030 (14)	0.0053 (13)
C7	0.0593 (15)	0.0486 (15)	0.0545 (17)	0.0003 (13)	−0.0022 (15)	0.0026 (13)
C8	0.0568 (15)	0.0628 (18)	0.0488 (16)	0.0107 (14)	−0.0024 (14)	0.0000 (13)
C9	0.0695 (18)	0.0541 (16)	0.0502 (17)	0.0128 (15)	−0.0010 (15)	0.0031 (14)
C10	0.0630 (16)	0.0529 (16)	0.0465 (15)	0.0143 (14)	0.0030 (14)	0.0079 (13)
C11	0.077 (2)	0.068 (2)	0.065 (2)	0.0228 (17)	0.0037 (17)	−0.0005 (15)
C12	0.078 (2)	0.088 (3)	0.073 (2)	0.028 (2)	0.0169 (19)	0.0067 (19)
C13	0.0607 (18)	0.098 (3)	0.084 (2)	0.014 (2)	0.0059 (19)	0.020 (2)
C14	0.069 (2)	0.095 (3)	0.068 (2)	−0.0010 (19)	−0.0033 (18)	−0.0019 (18)
C15	0.0646 (18)	0.075 (2)	0.0536 (18)	0.0085 (16)	0.0027 (16)	−0.0027 (15)
C16	0.0499 (15)	0.0567 (17)	0.061 (2)	−0.0012 (13)	−0.0040 (15)	−0.0023 (15)

### Geometric parameters (Å, º)

**Table:**

Cl1—C3	1.734 (3)	C7—C16	1.474 (4)
O1—C9	1.216 (3)	C8—H8A	0.9700
N1—C16	1.134 (4)	C8—H8B	0.9700
C1—H1	0.9300	C8—C9	1.512 (4)
C1—C2	1.385 (4)	C9—C10	1.489 (4)
C1—C6	1.382 (4)	C10—C11	1.383 (4)
C2—H2	0.9300	C10—C15	1.379 (4)
C2—C3	1.369 (4)	C11—H11	0.9300
C3—C4	1.379 (4)	C11—C12	1.378 (4)
C4—H4	0.9300	C12—H12	0.9300
C4—C5	1.379 (4)	C12—C13	1.368 (5)
C5—H5	0.9300	C13—H13	0.9300
C5—C6	1.376 (4)	C13—C14	1.378 (4)
C6—C7	1.518 (4)	C14—H14	0.9300
C7—H7	0.9800	C14—C15	1.380 (5)
C7—C8	1.533 (4)	C15—H15	0.9300
			
C2—C1—H1	119.5	H8A—C8—H8B	107.9
C6—C1—H1	119.5	C9—C8—C7	112.4 (2)
C6—C1—C2	120.9 (3)	C9—C8—H8A	109.1
C1—C2—H2	120.2	C9—C8—H8B	109.1
C3—C2—C1	119.6 (3)	O1—C9—C8	119.8 (3)
C3—C2—H2	120.2	O1—C9—C10	120.4 (3)
C2—C3—Cl1	120.4 (3)	C10—C9—C8	119.8 (2)
C2—C3—C4	120.3 (3)	C11—C10—C9	118.7 (3)
C4—C3—Cl1	119.3 (2)	C15—C10—C9	122.9 (3)
C3—C4—H4	120.2	C15—C10—C11	118.4 (3)
C5—C4—C3	119.6 (3)	C10—C11—H11	119.5
C5—C4—H4	120.2	C12—C11—C10	121.1 (3)
C4—C5—H5	119.5	C12—C11—H11	119.5
C6—C5—C4	121.1 (3)	C11—C12—H12	120.1
C6—C5—H5	119.5	C13—C12—C11	119.8 (3)
C1—C6—C7	122.0 (2)	C13—C12—H12	120.1
C5—C6—C1	118.5 (3)	C12—C13—H13	120.0
C5—C6—C7	119.5 (3)	C12—C13—C14	120.0 (3)
C6—C7—H7	107.4	C14—C13—H13	120.0
C6—C7—C8	112.8 (2)	C13—C14—H14	120.0
C8—C7—H7	107.4	C13—C14—C15	119.9 (3)
C16—C7—C6	111.8 (2)	C15—C14—H14	120.0
C16—C7—H7	107.4	C10—C15—C14	120.8 (3)
C16—C7—C8	109.7 (2)	C10—C15—H15	119.6
C7—C8—H8A	109.1	C14—C15—H15	119.6
C7—C8—H8B	109.1	N1—C16—C7	178.9 (3)
			
Cl1—C3—C4—C5	179.0 (2)	C6—C1—C2—C3	−0.4 (4)
O1—C9—C10—C11	7.2 (4)	C6—C7—C8—C9	−166.1 (2)
O1—C9—C10—C15	−172.7 (3)	C7—C8—C9—O1	4.4 (4)
C1—C2—C3—Cl1	−179.0 (2)	C7—C8—C9—C10	−175.9 (2)
C1—C2—C3—C4	1.8 (4)	C8—C9—C10—C11	−172.5 (2)
C1—C6—C7—C8	−75.1 (3)	C8—C9—C10—C15	7.6 (4)
C1—C6—C7—C16	49.1 (3)	C9—C10—C11—C12	−179.6 (3)
C2—C1—C6—C5	−1.0 (4)	C9—C10—C15—C14	179.6 (3)
C2—C1—C6—C7	177.1 (3)	C10—C11—C12—C13	−0.1 (5)
C2—C3—C4—C5	−1.8 (4)	C11—C10—C15—C14	−0.3 (4)
C3—C4—C5—C6	0.4 (4)	C11—C12—C13—C14	−0.1 (5)
C4—C5—C6—C1	1.0 (4)	C12—C13—C14—C15	0.1 (5)
C4—C5—C6—C7	−177.2 (2)	C13—C14—C15—C10	0.1 (5)
C5—C6—C7—C8	103.0 (3)	C15—C10—C11—C12	0.3 (4)
C5—C6—C7—C16	−132.8 (3)	C16—C7—C8—C9	68.5 (3)
